# Guyon's canal resulting from lipoma: A case report and review of the literature

**DOI:** 10.1016/j.ijscr.2022.107182

**Published:** 2022-05-11

**Authors:** Vinh Pham Quang, Huy Hoang Quoc, Bach Nguyen, Chuong Ngo Quang, Hieu Nguyen Chi, Ngoc Nguyen

**Affiliations:** a Department of Orthopaedics and Rehabilitation, University of Medicine and Pharmacy at Ho Chi Minh City, 217 Hong Bang Street, District 5, Ho Chi Minh City, Viet Nam; b Department of Orthopaedics, University Medical Center Ho Chi Minh City, 201 Nguyen Chi Thanh Street, District 5, Ho Chi Minh City, Viet Nam

**Keywords:** Guyon's canal syndrome, Lipoma

## Abstract

**Introduction:**

Compression of the ulnar nerve at the wrist is a rare condition. Clinical presentations of ulnar nerve compression at the wrist and hand can vary widely due to the complex anatomic course of ulnar nerve at the wrist. Lipoma is a common benign soft tissue tumor and rarely causes neuropathy. We present a rare case of Guyon's canal syndrome caused by lipoma.

**Case reports:**

We describe a case with acute ulnar nerve deficit. Clinical examination, EMG and MRI suggested Guyon's canal syndrome. Surgical exploration revealed a well-encapsulated fatty tumor within Guyon's canal. Histopathology demonstrated mature adipose tissue consistent with lipoma. Post-operative course was uneventful and the sensory symptom had subsided at the three month follow up.

**Discussion and conclusion:**

There are many etiologies of Guyon's canal syndrome which can be classified into groups. Symptoms of ulnar nerve compression at the wrist can vary depend on the location of the lesion. Treatment options depend on severity and duration of symptoms, previous treatment attempts, and underlying etiology. Lipoma is not a common cause of Guyon's canal with few case reports in the literature. Surgery to open and release the roof of Guyon's canal and excision of the lipoma can yield good outcome in most reports.

## Highlights

• Guyon's canal syndrome can be caused by a variation of etiology which compress and damage the ulnar nerve.
• Guyon's canal syndrome caused by lipoma is an extremely rare condition.
• Nonoperative measures can give positive result in early stage or in some circumstances.
• Surgical treatment can achieve a good outcome, especially when underlying etiology is tumors group (lipoma in our case).

## 1 Introduction and importance

Guyon's canal syndrome is a relatively rare condition. This disorder is caused by compression of the ulnar nerve when it passes through the Guyon's canal. The wrist is the second most common site of ulnar nerve entrapment following the elbow. Diagnosis of Guyon's canal syndrome is usually based on clinical examination. However, due to the variation of clinical presentations, the clinical diagnosis is not always straightforward, particularly when the sensory symptoms are atypical or absent. Moreover, it is usually difficult to localize the compression site along nerve course based on clinical examination or electrophysiology.

Lipoma is one of the most common benign tumor that is composed of mature adipocytes. Lipoma rarely causes neuropathy. Very few cases of ulnar nerve entrapment resulting from lipoma in the Guyon's canal have been reported in the literature. We report a rare case of acute ulnar nerve neuropathy caused by a lipoma at the Guyon's canal level.

## 2 Case presentation

A 35-year-old male sawyer presented with burning pain and numbness along the little finger and ulnar aspect of the ring finger of the left hand for 3 months. He complained of difficulty in holding tools at work due to left hand pain. He does not have any drug and family history relevant to his condition. On examination, there was marked atrophy of the dorsal interosseous, palmer interosseous, abductor digiti minimi, and abductor pollicis muscle. Wartenberg sign was positive, Froment test was positive in the left hand, and Tinel sign was positive at the level of Guyon's canal.

Conduction of velocity studies of the left ulnar nerve showed prolonged distal sensory latency of 2.71 ms (compared to 3.5 ms in the right hand), decreased sensory velocity (37 m/s in the left hand vs. 45 m/s in the right hand), reduced amplitude of the interosserous muscle, and lowered conduction velocity of the motor nerve at the wrist. Electrodiagnostic studies suggest compression of the ulnar nerve at the Guyon's level.

**Fig. 1:**
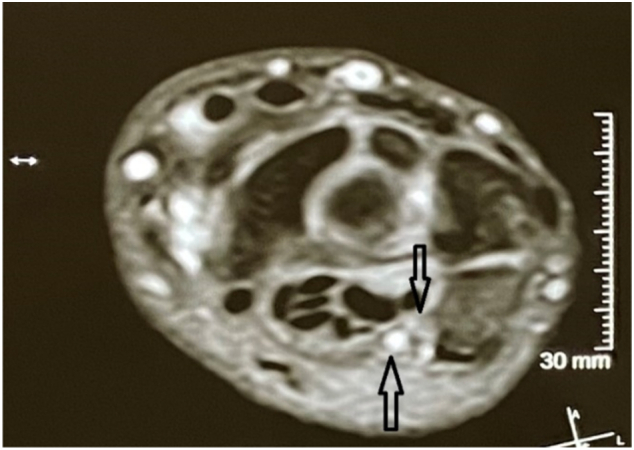
MRI show Guyon's canal with a mass inside.

Magnetic resonance imaging (MRI) revealed a small lesion in Guyon's canal next to the ulnar nerve. The Ulnar nerve showed hyper-intensity in T2-signal (Fig. 1).

**Fig. 2:**
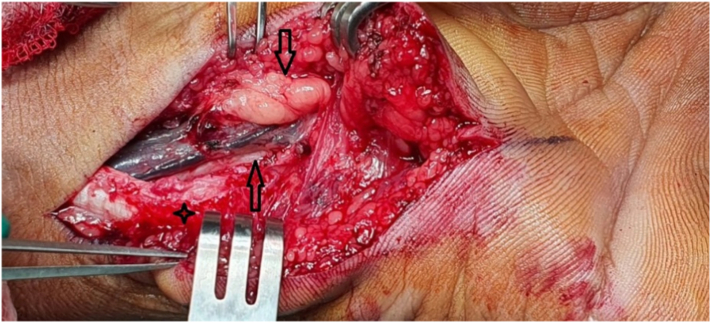
Intra-operative picture show lipoma in Guyon's canal which compress ulnar nerve.

**Fig. 3:**
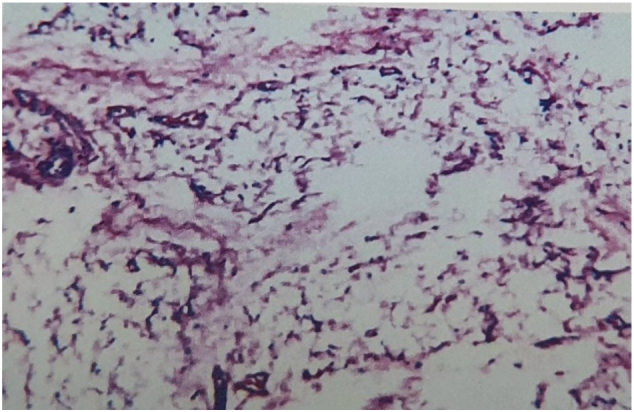
Microscopically, the tumor was composed of uniform, well-differentiated mature adipocytes with small nuclei and abundant clear cytoplasm. Method: This case report is compliant with the SCARE Guideline 2020 [1].

The surgery was done by the first author with experience in peripheral nerve disorder. Patient was prepared for the surgery. On surgical table, patient was on supine position and tourniquet was used. Exploratory surgery was performed. Following incision of the carpal ligament, a lobulated, bluish yellow mass protruded. The mass measuring 1,5 × 1 × 2 cm, which had been compressing the ulnar artery and the ulnar nerve, was completely excised. Histopathology showed mature adipose tissue consistent with lipoma. Postoperative course was uneventful with progressive resolution of numbness and pain. He was under non-restrictive rehabilitation postoperatively. The patient was pleased with the result (Fig. 2, Fig. 3).

## 3 Clinical discussion

### 3.1 Guyon's canal anatomy

The Guyon's canal, named after a French surgeon Felix Guyon, is a fibro-osseous tunnel within the ulnar side of the wrist. In general, the Guyon's canal is bounded [2], [3], [4]:

• Radially by the hook of hamate, the flexor tendons, and the transverse carpal ligament
• Ulnarly by the pisiform, the tendon of the flexor carpi ulnaris, and the abductor digiti minimi
• Volarly by the palmar carpal ligament, the palmaris brevis, and the hypothenar connective tissue.
• Dorsally by the transverse carpal ligament, the piso-hamate ligament, the piso-metacarpal ligament, tendons of the flexor digitorum profundus, and the opponens digiti minimi.

### 3.2 Etiology of Guyon's canal syndrome

There are many conditions that cause Guyon's canal syndrome which can be categorized into 7 groups [5]: (1) Tumors (e.g. ganglion cyst, lipoma, etc.); (2) repetitive trauma (e.g. hypothenar syndrome); (3) adjacent vascular enlargement (e.g. ulnar artery aneurysm); (4) metabolic diseases; (5) variation of Guyon's canal structure (e.g. piso-hamate arch); (6) wrist degeneration; (7) idiopathic conditions. Among these causative factors, ganglion cyst is the most common cause of Guyon's canal syndrome.

### 3.3 Classification of ulnar neuropathy in the Guyon's canal

Inside the Guyon's canal, the ulnar nerve is a mixed nerve which divides into superficial sensory and deep motor branches along its course. Therefore, Shea and McClain classified ulnar lesions based on affected regions which can be defined as 3 zones [6].

Zone 1: The proximal part of the ulnar nerve inside the Guyon's canal: proximal to the motor/sensory bifurcation, causing both motor and sensory symptoms.

Zone 2: Distal to the bifurcation and affecting deep motor branches only, causing motor deficits and muscle atrophy. This is the most commonly affected region.

Zone 3: Distal to the bifurcation and affecting the superficial sensory fibers only, causing sensory symptoms

### 3.4 Lipoma in the Guyon's canal

Lipoma is a benign soft tissue tumor that composed of mature adipocytes. With only few case reports, it is thought to be a very rare causative factor that results in narrowing of the Guyon's canal [7]. The reported size of the tumor that compressed the canal varied from the 1 × 1.5 cm (area) to 6.5 cm × 4 cm × 2.5 cm (volume). In our case, the lipoma size is 1,5 × 1 × 2 cm which is difficult to identified on wrist MRI. In all cases, including ours, resection of the lipoma resection led to alleviation of symptoms upon follow up.

### 3.5 Treatment options

Treatments of Guyon' canal syndrome depend on the severity of the symptoms (numbness, pain, weakness, and muscle atrophy), duration of the symptoms (acute, sub-acute or chronic), previous treatments given, and underlying etiology. According to P. Hoogvliet, patients with mild or moderate symptoms with a duration of less than three months can be treated non-operatively. Patients should be instructed to avoid activities that apply pressure on the Guyon's canal, such as lifting weights or bicycling, and to limit mechanical overload that can result from repetitive movements or static postures such as prolonged extension of the wrist. Neutral splint with the free fingers can be used at night for 1 to 12 weeks. NSAIDs and corticosteroid injections are not beneficial for the treatment of Guyon's canal syndrome [8], [9]. Surgical treatment is indicated for patients with severe symptoms that last more than 3 months. Surgery is also indicated for patients with mechanical compression of the Guyon's canal such as ganglion cyst or tumor, and those that did not respond to initial, non-operative treatments. The aim of the surgery is to reduce the pressure on the ulnar nerve in the Guyon's canal by opening the roof of Guyon' canal and removing all structures that compress the nerve, thereby reducing the symptoms of Guyon's canal syndrome [10].

### 3.6 Surgical approach

Many surgical approaches can be used such as (1) carpal tunnel incision (longitudinal, extended proximally across the wrist flexion crease with a transverse component at the wrist crease), (2) ulnar hypothenar approach, Brunner approach and (3) the ulnar hypothenar approach, longitudinal. There are no consensus on the best surgical approach for the Guyon's canal release [9]. After identification and incision of the volar carpal ligament, the bifurcation point of the ulnar nerve should be located. All potentially compressive structures such as mass, tight bands, tendinous arcades, or anomalous muscles constricting along the nerve and its branch should be meticulously released. Post-operative care includes hand elevation, appropriate rest of the hand, and restriction of heavy lifting until the suture is removed. Physical therapy to promote nerve gliding and muscles strength of the hand is indicated. Splint is not routinely but can be prescribed in patients who have a tendency to put mechanical load on the Guyon's canal [11], [12].

## 4 Conclusion

Guyon' canal syndrome is less common than either carpal tunnel syndrome or cubital tunnel syndrome. The anatomy of the ulnar tunnel is complex. MRI appears to be important for the diagnosis of patients with symptoms of ulnar nerve compression to for identifying the underlying etiology preoperatively. Lipoma is a benign soft tumor that rarely compress surrounding structures. However it can cause compression when occur in a closed compartment such as the Guyon' canal. Surgical removal of lipoma can yield excellent results, although at this time, case series and comparative studies are uncommon because of the rarity of this condition.

## Sources of funding

None.

## Ethical approval

This case series got ethical approval from our institution. The patient was given consent form before the surgery.

## Consent

Written informed consent was obtained from the patient for publication of this case report and accompanying images. A copy of the written consent is available for review by the Editor-in-Chief of this journal on request.

## Research registration

None.

## Guarantor

Vinh Pham Quang MD, PhD.

## Provenance and peer review

Not commissioned, externally peer-reviewed.

## CRediT authorship contribution statement

*Vinh Pham Quang*: conceptualising the plan for surgery, performing the surgery, writing the literature review for case report, reviewing the manuscript.

*Huy Hoang Quoc*: Assisting in planning and in the surgery, writing the draft for case report.

*Bach Nguyen*: Assisting the surgery, writing the literature review.

*Chuong Ngo Quang*: taking note and data visualisation perioperatively.

*Hieu Nguyen Chi*: Assisting the surgery, prepare the necessary equipments.

*Ngoc Nguyen*: Analyzing the radiology and MRI.

## Declaration of competing interest

All authors state that there are no financial and personal relationships with other people or organizations that could inappropriately influence this case series.
